# Comparison between endoscopic mucosal resection with a cap and endoscopic submucosal dissection for rectal neuroendocrine tumors

**DOI:** 10.1186/s12893-022-01693-x

**Published:** 2022-06-27

**Authors:** Xiuli Zheng, Mingli Wu, Huihui Shi, Limian Er, Kan Wang, Ying Cao, Shengmian Li

**Affiliations:** 1 Department of Endoscopy, The Fourth Hospital of Hebei Medical University, No. 12 Jiankang Road, Chang ’an District, Shijiazhuang, 050000 Hebei China; 2 Department of Gastroenterology, The Fourth Hospital of Hebei Medical University, No. 12 Jiankang Road, Chang ’an District, Shijiazhuang, 050000 Hebei China

**Keywords:** Rectal neuroendocrine tumor, Endoscopic mucosal resection with a cap, Endoscopic submucosal dissection, Incomplete resection

## Abstract

**Background:**

The aim of this study is to evaluate and compare the safety and efficacy of endoscopic mucosal resection with a cap (EMR-c) with those of endoscopic submucosal dissection (ESD) for rectal neuroendocrine tumors (R-NETs) ≤ 15 mm in diameter, and to analyze the risk factors of incomplete resection.

**Methods:**

A total of 122 patients who underwent EMR-c or ESD for R-NETs at the Fourth Hospital of Hebei Medical University between February 2007 and December 2020 were invovled in this study. The clinical outcomes of two groups were compared and evaluated.

**Results:**

A total of 122 patients with 128 R-NETs underwent endoscopic resection (EMR-c, 80; ESD, 48). In terms of duration of operation, EMR-c was significantly shorter than ESD (*p* < 0.001). Univariate analysis and multivariate analysis suggested that tumor diameter ≥ 8 mm was an independent risk factor for incomplete resection in patients with R-NETs in this study.

**Conclusions:**

Both EMR-c and ESD were safe and effective treatments for R-NETs ≤ 15 mm in diameter. In addition, tumor diameter ≥ 8 mm was an independent risk factor for incomplete resection.

## Introduction

With the widespread use of screening colonoscopy, the incidence of rectal neuroendocrine tumors (R-NETs) has increased recently [1]. R-NETs account for up to 27% of gastroenteropancreatic NETs and 17.7% of all NETs in the SEERdatabase [1]. And R-NETs are reportedly more in Asian populations [2]. R-NETs can be found incidentally without symptoms and have good prognosis, but metastases can occur in some cases even with relatively small tumors [3, 4]. The prognosis for advanced R-NETs is similar to adenocarcinoma [3]. Hence, early detection and treatment are particularly important.

Endoscopic resection is recommended for small, non-lymph node metastatic R-NETs that invade the mucosa or submucosa. There are many methods of endoscopic resection, and different methods of endoscopic resection may be selected according to the habits of the surgeon and the limitations of equipment. Many literatures have described endoscopic mucosal resection with a cap (EMR-c) and endoscopic submucosal dissection (ESD) are two options of endoscopic resection for R-NETs [5–10]. However, the optimal strategy is poorly understood. Therefore, we compared the safety and efficacy of the two methods of endoscopic resection for R-NETs, and to analyze the risk factors of incomplete resection.

## Methods

### Patients

We collected the case characteristics and follow-up data of 122 patients with R-NETs who underwent resection in the Endoscopy Department of the Fourth Hospital of Hebei Medical University from February 2007 to December 2020. Inclusion criteria: patients underwent EMR-c or ESD; tumor size ≤ 15 mm. Exclusion criteria: (1) CT/MRI suggested lymphatic metastasis or distant metastasis; (2) complicated with other malignant tumors. We compared the duration of operation, complications, pathological types and clinical prognosis between the EMR-c group and the ESD group. The study was reviewed and approved by the Ethics Committee of the Fourth Hospital of Hebei Medical University (ID: 2021KS002).

### EMR-c procedure and ESD procedure

It has been reported in the literatures, endoscopic ultrasonography (EUS) is useful for evaluating preoperatively the size and invasion depth of rectal NETs [11–13]. Therefore, EUS (20 MHz, Olympus, Japan) was used to assess the size and depth of the lesion before surgery to select an appropriate surgical approach. All lesions in this study did not invade muscularis propria and the size was less than 15 mm. As EUS was not available before 2010, 18 patients with18 lesions did not undergo preoperative ultrasound endoscopy. EMR-c procedure and ESD procedure, therapeutic endoscope with water supply function (GIF-H260J, Olympus, Japan) and high-frequency electric therapy instrument (Alerbo, Germany) were adopted. All surgeries were performed by four professional endoscopists who had worked for more than 7 years.

**Fig. 1:**
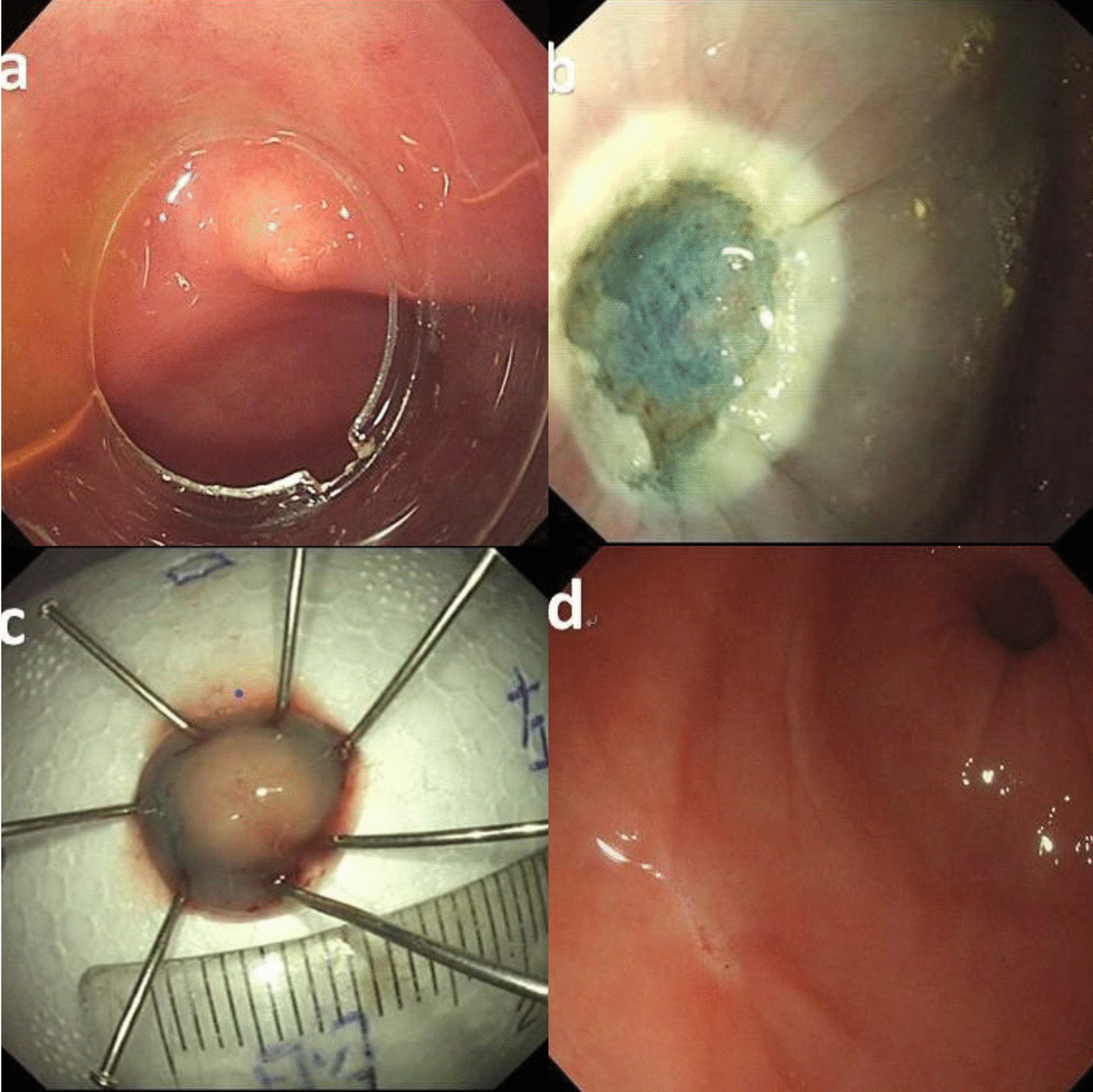
Endoscopic mucosal resection with a cap (EMR-c): **a** transparent cap is attached to the distal end of the scope; **b** a clear resection surface is observed; **c** the resection specimen is retrieved and measured; **d** scarring had formed after 6 months

For EMR-c (Fig. 1), a wide (14.9 mm-diameter), transparent cap (D-201-11802, Olympus, Japan) was fitted to the tip of endoscope. The capped endoscope was placed over the lesion, followed by suction to engulf the lesion inside the cap, and a snare was then used to remove the lesion.

**Fig. 2:**
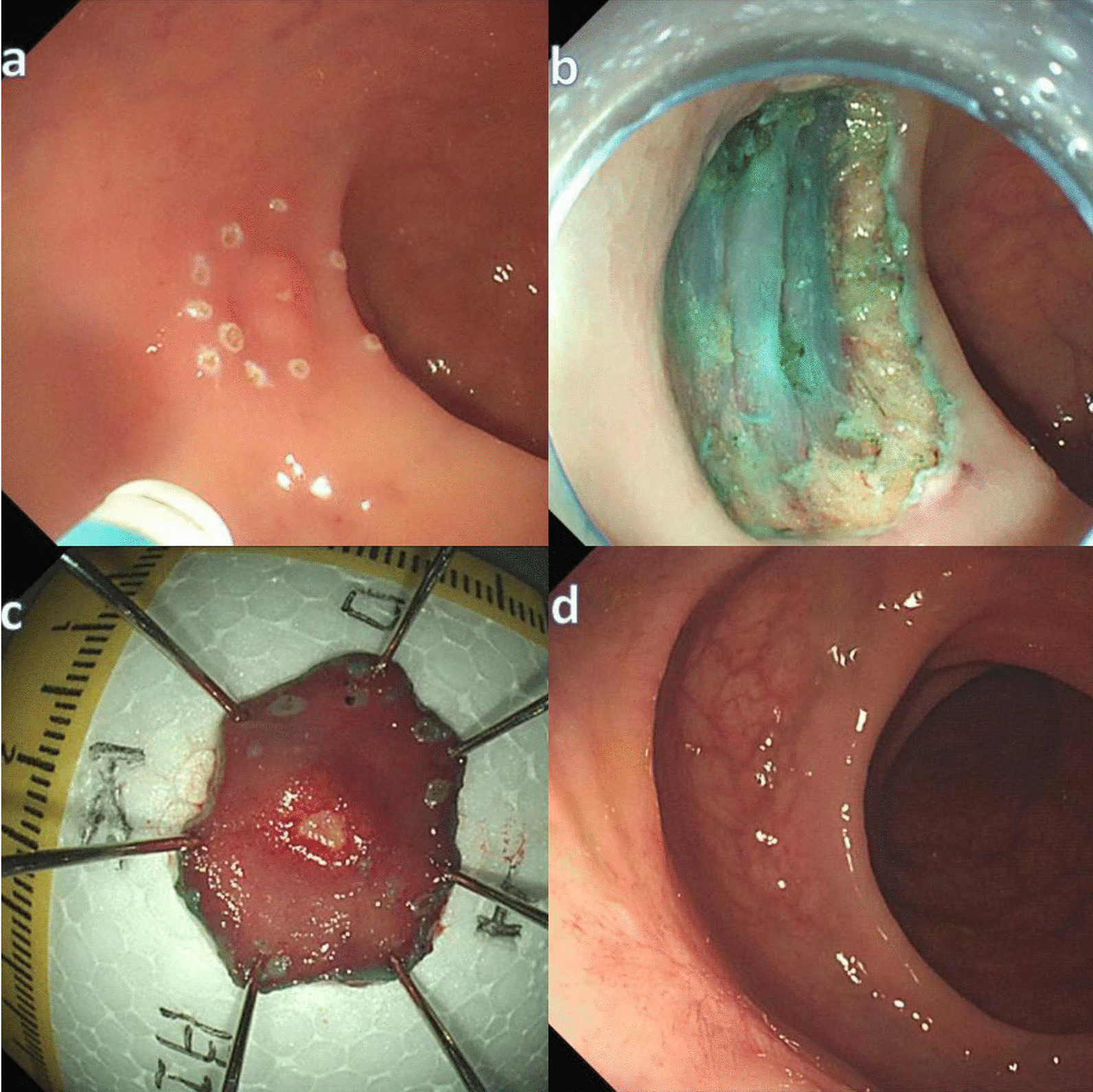
Endoscopic submucosal dissection (ESD): **a** labeled around the tumor; **b** a clear resection surface is observed; **c** the resection specimen is retrieved and measured; **d** scarring had formed after 6 months

For ESD (Fig. 2), a short transparent cap (D-201-11804, Olympus, Japan) was attached to the tip of endoscope. The lesion boundary was marked, the submucosal solution was injected, the circumferential mucosa of the lesion was incised using a dual knife (KD-650Q, Olympus, Japan), and the lesion was gradually exfoliated.

### Histological evaluation

Resected specimens were fixed to foam board with a ruler and then soaked in formalin solution. The histopathological evaluation included the histological type, lateral and vertical resection margins, and invasion layer, which was determined by hematoxylin & eosin and immunohistochemical staining in line with the 2019 World Health Organization classification of tumors of the digestive system. All sections were evaluated by experienced pathologists. En bloc resection was defined as the resection of the entire tumor in a single piece, as observed endoscopically. A pathologically complete resection (P-CR) was defined as an endoscopic resection with no lateral or vertical margins of the tumor. Incomplete resection was considered to have occurred when a resection failed to achieve tumor-free margins.

### Complications

Postoperative bleeding and perforation are the two most common complications after endoscopic resection. Postoperative bleeding was determined when postoperative hematochezia occurs, requiring endoscopic hemostasis. Perforation was defined as a breakdown of the muscularis propria, postoperative abdominal pain, or the presence of gas in the abdominal cavity indicated by imaging examination.

### Follow-up

Postoperative routine colonoscopy and CT were performed at 3 months, 6 months and 12 months to observe whether there was recurrence or metastasis. Colonoscopy and CT were recommended every 2 years thereafter. The deadline for our follow-up was September 2021.

### Statistical analysis

Continuous variables are presented as the mean with standard deviation or the median with range and were evaluated by the t-test. Categorical data are expressed as numbers and percentages, and analysis was conducted through a chi-square test. Binary Logistic regression analysis was used for multivariate analysis for factors related to incomplete resection. Statistical analysis was performed using IBM SPSS Statistics v.25.0.0 (IBM Corp, New York), and *p* < 0.05 (two-tail) was considered statistically significant.

## Results

In this study, we enrolled 122 patients with 128 lesions for analysis, including 76 patients receiving EMR-c and 46 patients receiving ESD for R-NETs resection. Preoperative evaluations were T1N0M0. Table 1 shows the patients’ baseline characteristics.

**Table 1:** Baseline characteristics of patients and tumors

	EMR-c	ESD	*p*-value
Patient characteristics			
Patient number, n (%)	76	46	
Age, years			
Mean ± SD	52.39 ± 11.958	51.37 ± 10.186	0.629
Gender, n (%)			
Male	47 (61.84)	24 (52.17)	
Female	29 (38.16)	22 (47.83)	0.345
With single/multiple lesions, n (%)			
Single	72 (94.74)	44 (95.65)	
Multiple	4 (5.26)	2 (4.35)	1.000
Lesion characteristics			
Lesion number, n (%)	80	48	
Lesion size, mm			
Mean ± SD	6.988 ± 2.357	6.438 ± 1.988	0.179
Lesion size group, n (%)			
< 8 mm	46 (57.50)	29 (60.62)	
≥ 8 mm	34 (42.50)	19 (39.58)	0.853
Location group, n (%)			
Upper rectum	4 (5.00)	1 (2.08)	
Middle rectum	50 (62.5)	24 (50.00)	
Lower rectum	26 (32.5)	23 (47.82)	0.223
Histopathological grade, n (%)			
G 1	62 (93.94)	42 (89.36)	
G 2	4 (6.06)	5 (10.64)	0.486

*EMR-c* endoscopic mucosal resection with a cap, *ESD* endoscopic submucosal dissection

There were no statistically significant differences in age, gender, lesion size, lesion location and pathological grade between the EMR-c group and the ESD group. There were more males than females in the two groups (71/51). Among them, 58.59% of the lesions were smaller than 8 mm, 96.09% of the lesions were located in the middle and lower rectum, and the size of EMR-c group and ESD group were 6.988 ± 2.357 mm and 6.438 ± 1.988 mm, respectively. And 104 patients with 110 lesions were examined by EUS, and all lesions were located in the mucosal muscular layer and submucosal layer. Immunohistochemical examination of 113 lesions indicated that 104 lesions (92.04%) were G1.

Of the 128 lesions, 80 were excised by EMR-c and 48 by ESD (Table 2). In terms of duration of operation, EMR-c was significantly shorter than ESD (10.100 ± 2.096 min vs. 28.688 ± 4.172 min, *p* < 0.001). There was no significant difference in EMR-c and ESD between the two groups in terms of En bloc resection rate (97.5% vs. 100% respectively), P-CR rate (85% vs. 77.08%, respectively), and complication rate of 2.5% for both. Postoperative bleeding occurred in two lesions in EMR-c group, and titanium clips were used for hemostasis. Perforation occurred only in the ESD group, and only in 2 cases, and conservative treatment was effective after titanium clips were closed.

**Table 2:** Clinical outcomes of EMR-c and ESD

Factors	EMR-c	ESD	*p*-value
Number	80	48	
Duration of procedure, min			
Mean ± SD	10.100 ± 2.096	28.688 ± 4.172	< 0.001
En bloc resection, n (%)	78 (97.50)	48 (100.00)	0.528
P-CR rate, n (%)	66 (84.62)	37 (77.08)	0.345
Vertical positive	10	11	N/A
Lateral positive	2	0	
Complication	2 (2.50)	2 (4.17)	0.276
Postprocedural bleeding	2	0 (0.00)	N/A
Perforation	0 (0.00)	2	

*EMR-c* endoscopic mucosal resection with a cap, *ESD* endoscopic submucosal dissection

Among them, 23 lesions were not completely resected, 2 lesions were positive for lateral resection margins, and 21 lesions were positive for vertical resection margins. Only one patient underwent additional surgery, and no residual tumor was found in surgical pathology.

During follow-up, 32 patients (32 lesions) were lost to follow-up. A total of 90 patients with 96 lesions were followed up in this study, including 52 patients in the EMR-c group and 38 patients in the ESD group. The median follow-up time was 36 months (range 9–120 months). There were no local recurrences or metastasis in any patients in either group during the follow-up period.

In the analysis of pathological features of the complete resection group and the incomplete resection group (Table 3), the difference in the lesion size between the two groups was statistically significant (*p* = 0.018). Multivariate analysis (Table 4) showed that tumor size ≥ 8 mm (OR: 3.419, 95% CI 1.295–9.026, *p* = 0.013) was independent risk factor for incomplete resection in patients with R-NETs in this study.

**Table 3:** Clinicopathological features of complete and incomplete resection

Factors	Complete resection (n = 105)	Incomplete resection (n = 23)	*p*-value
Lesion size group, n (%)			
< 8 mm	67	7	
≥ 8 mm	38	16	0.018
Location group, n (%)			
Upper rectum	4	1	
Middle rectum	59	15	
Lower rectum	42	7	0.592
Histopathological grade, n (%)			
G 1	83	21	
G 2	7	2	1.000
Operation, n (%)			
EMR-c	68	12	
ESD	37	11	0.342

*EMR-c* endoscopic mucosal resection with a cap, *ESD* endoscopic submucosal dissection

**Table 4:** Multivariate analysis of risk factors for positive resection margin of R-NETs

Factors	OR	95% CI	*p*-value
Lesion size group, n (%)			
< 8 mm	1	Reference	
≥ 8 mm	3.419	1.295–9.026	0.013
Location group, n (%)			
Upper rectum	1	Reference	
Middle rectum	0.635	0.061–6.639	0.704
Lower rectum	0.410	0.036–4.700	0.474
Operation, n (%)			
EMR-c	1	Reference	
ESD	2.010	0.759–5.323	0.160

*OR* odds ratio, *95% CI* 95% confidence interval, *EMR-c* endoscopic mucosal resection with a cap, *ESD* endoscopic submucosal dissection

## Discussion

R-NETs commonly appear as submucosal lesions with yellowish mucosa [14, 15]. There is still controversy on the treatment of R-NETs, and numerous treatment strategies have been reported [7, 16], including EMR, EMR-c, EMR-l, ESD, etc. In this study, we retrospectively analyzed the safety and effectiveness of EMR-c and ESD in R-NETs ≤ 15 mm, and predicted the risk factors of incomplete resection. The results showed that both EMR-c and ESD were safe and effective for R-NETs resection. The P-CR rate and complication rate were similar between the two groups, and the duration of operation was significantly shorter in EMR-c group than in ESD group. Furthermore, it was proposed that tumor larger than 8 mm was an independent risk factor for incomplete resection.

EMR-c has been widely used for small R-NETs due to its operability and short duration. However, its use has aroused great controversy in previous studies [6, 17–21]. The P-CR rate has been reported to range from 50 to 100%, probably because of the small sample size. In our study, 80 lesions were resected by EMR-c, and the P-CR rate was 85%, which was not significantly different from that (77.08%) in the ESD group. Wang [9] compared the therapeutic effect of EMR-c and ESD on R-NETs with a diameter less than 16 mm, and the P-CR rate of EMR-c group was 83.3%, which was consistent with our results. The higher P-CR rate of EMR-c may be due to the fact that CAP provides a sufficient vertical distance to excise the lesion. ESD is widely recommended because of its higher resection rate. Chen [22] conducted a retrospective study of 66 patients with R-NETs less than 15 mm in diameter, and the results showed that the P-CR rate was 96.43% for ESD. In this study, the duration of operation in the ESD group was relatively longer, with a P-CR rate of merely 77.08%. In addition, there were 2 cases of perforation, which might be attributed to two factors: the lesions that were located in the deep submucosal layer, and the different technical level of the operator. This indicates that ESD operation has a higher requirement for operators and a longer learning cycle. In order to prevent perforation, we can use titanium clip to seal the wound as an added precaution. This study showed that compared with ESD, EMR-c was simpler and the duration of operation was shorter, and this method is preferable for doctors in primary hospitals and less experienced/skilled doctors.

In this study, 23 lesions were not completely resected, among which 21 lesions were positive for vertical resection margins. We analyzed the relevant factors, which indicated that tumor size ≥ 8 mm was independent risk factor for incomplete resection. It suggests that lesions ≥ 8 mm may involve the submucosal depth, which require us to remove the lesions close to the muscular layer, or even remove part of the muscular layer. In previous studies, R-NETs could not be resected radically, and its influencing factors might include tumor size [23, 24], central depression [25], lesion morphology [26], pathological level [8], distance from anus [5], treatment methods [15], etc. However, incomplete resection does not necessarily indicate residual or recurrent tumors [6, 25]. In our study, only one of the 23 patients who did not receive complete resection was treated with additional surgery, and no tumor was found in the resected specimen, perhaps the electric cautery used in endoscopic resection could destroy the tumor margins [6]. The remaining 22 patients were followed up, and no recurrence or metastasis was reported to date.

In recent years, an underwater endoscopic mucosal resection (UEMR) has been used to remove small R-NETs [27, 28]. This method eliminates submucosal injection and may increase the buoyancy of submucosal tumors, which lift and float away from the muscularis propria [28]. In Sung Sil Park’s study [27], 36 patients were treated with UEMR and 79 with ESD. There was no difference in P-CR rate between the two groups, but the operative time of the UEMR group was significantly shorter than that of the ESD group. However, UEMR has its limitations [27]. Patients with inadequate intestinal preparation may not be able to identify lesions well. In future studies, we can try to evaluate the advantages and disadvantages of these methods comprehensively.

The study has several limitations. First, this study is a single-center retrospective study, which may cause selection bias. Second, the surgical procedure is not performed by the same physician, so differences between different people are inevitable. In our recent study [29], tumor size greater than 15 mm was considered to be an independent risk factor for lymph node metastasis in patients with colorectal neuroendocrine neoplasm, so 15 mm was used as the cut-off value to study the difference in endoscopic resection methods. We noticed that a recent report [30] suggested that tumor size greater than 11.5 mm was independent risk factor for lymph node metastases in patients with R-NETs. Maybe it's because of the different databases or the differences between the eastern and western populations. Therefore, a well-designed, multi-center, multi-site clinical study is needed to confirm these conclusions in the future.

## Conclusions

Both EMR-c and ESD were safe and effective treatments for R-NETs ≤ 15 mm in diameter. The findings in this study suggested that tumor diameter ≥ 8 mm was an independent risk factor for incomplete resection in patients with R-NETs.

## Data Availability

The datasets used and analyzed during the current study are available from the corresponding author upon reasonable request without breaching participant confidentiality.

## Abbreviations

R-NETs: Rectal neuroendocrine tumors
EMR-c: Endoscopic mucosal resection with a cap
ESD: Endoscopic submucosal dissection
P-CR: Pathologically complete resection
OR: Odds ratio
95% CI: 95% Confidence interval
UEMR: Underwater endoscopic mucosal resection
